# Metal Release from Manganese Nodules in Anoxic Seawater and Implications for Deep-Sea Mining Dewatering Operations

**DOI:** 10.1021/acsestwater.4c00166

**Published:** 2024-06-27

**Authors:** Yang Xiang, Janelle M. Steffen, Phoebe J. Lam, Amy Gartman, Kira Mizell, Jessica N. Fitzsimmons

**Affiliations:** † Department of Ocean Sciences, University of California, Santa Cruz, California 95064, United States; ‡ Department of Oceanography, Texas A&M University, College Station, Texas 77840, United States; § U.S. Geological Survey, Pacific Coastal and Marine Science Center, Santa Cruz, California 95060, United States

**Keywords:** Deep-sea mining, polymetallic nodules, oxygen deficient zones, trace metals, reductive dissolution

## Abstract

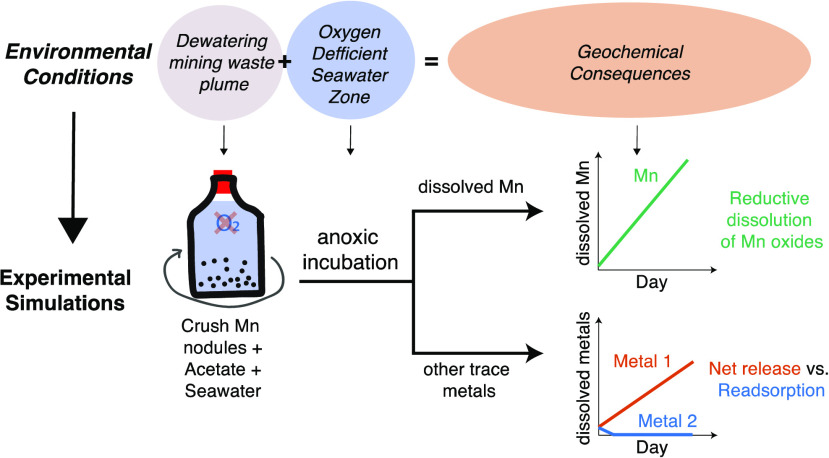

The potential mining of deep-sea polymetallic nodules has been gaining increasing attention due to their enrichment in metals essential for a low-carbon future. To date, there have been few scientific studies concerning the geochemical consequences of dewatered mining waste discharge into the pelagic water column, which can inform best practices in future mining operations. Here, we report the results of laboratory incubation experiments that simulate mining discharge into anoxic waters such as those that overlie potential mining sites in the North Pacific Ocean. We find that manganese nodules are reductively dissolved, with an apparent activation energy of 42.8 kJ mol^–1^, leading to the release of associated metals in the order manganese > nickel > copper > cobalt > cadmium > lead. The composition of trace metals released during the incubation allows us to estimate a likely trace metal budget from the simulated dewatering waste plume. These estimates suggest that released cobalt and copper are the most enriched trace metals within the plume, up to ∼15 times more elevated than the background seawater. High copper concentrations can be toxic to marine organisms. Future work on metal toxicity to mesopelagic communities could help us better understand the ecological effects of these fluxes of trace metals.

## Abstract 2

Geochemical consequences of proposed deep-sea mining waste discharge into oxygen-deficient zones are not well-understood. This study finds elevated copper levels in a simulated waste plume, which could be toxic to mesopelagic communities.

## Introduction

Marine polymetallic nodules in certain abyssal regions, also known as manganese (Mn) nodules, have attracted interest over several decades from mining companies due to their enrichment of metals such as cobalt (Co), nickel (Ni), copper (Cu), and rare earth elements (REEs).^1−3^ These nodules grow up to 20 cm in their longest dimension and are formed by the slow growth of Mn and iron (Fe) oxides (dominantly δ-MnO_2_, vernadite, and todorokite) around a nucleus at a rate of several millimeters to centimeters per million years.^4−6^

A significant increase in demand for minerals and metals, such as lithium (Li), Cu, Co, Ni, and REEs, is predicted in order to build the batteries required for the clean energy transition to a low-carbon future.^7−10^ As the proposed demand for these metals is likely to double by 2060,^11,12^ deep-sea mining of Mn nodules is an emergent industry that may provide additional mineral resources beyond the growth of land-based mining to meet this proposed demand.

**Figure 1:**
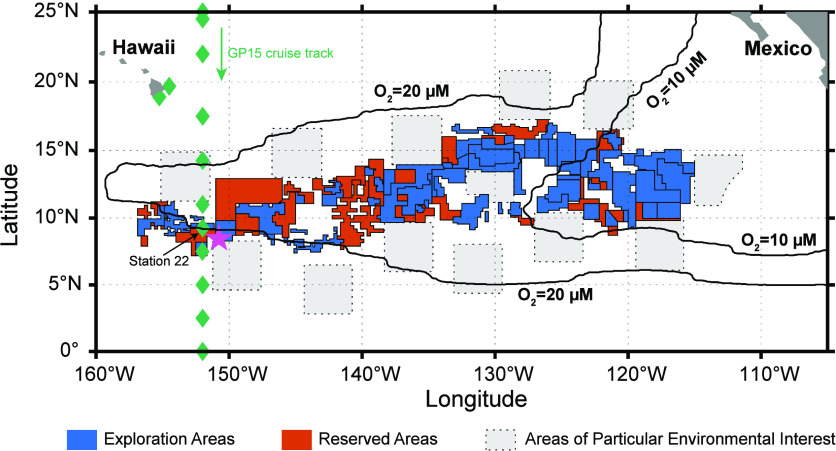
Map of the Clarion-Clipperton Zone (CCZ) in the Northeast Pacific Ocean. Blue and vermilion boxes are exploration and reserved areas, respectively. Light gray squares are areas of particular environmental interest (APEIs) designated by the ISA. The bluish-green diamonds are the U.S. GEOTRACES GP15 cruise track, and the location of Station 22, where the filtered seawater used for the incubation study was collected, is labeled. The location of Site A where the Mn nodules used in this study were collected is marked by using a star. The thick black contour lines are dissolved oxygen concentrations of 10 and 20 μM at the 500 m isobath, which were retrieved from the World Ocean Atlas 2018,^32^ illustrating the horizontal extent of the oxygen deficient zones (ODZs). Shapefiles are downloaded from the International Seabed Authority Web site (https://www.isa.org.jm/minerals/maps).

Mn nodules are widely distributed in the global ocean.^13^ One of the most extensive deposits of Mn nodules is the Clarion-Clipperton Zone (CCZ) in the Eastern Tropical North Pacific Ocean, an area between Hawaii and Mexico at ∼8–15° N. The CCZ is located beneath one of the world’s most extensive oxygen-deficient zones (ODZs), where waters between 200–800 m are generally lower than 20 μM (Figure 1) of oxygen and reach anoxic conditions in some areas. As interests in mining escalate, the International Seabed Authority (ISA) has issued exploration contracts to 17 countries and commercial entities in the CCZ (https://www.isa.org.jm/exploration-contracts). To date, no mining, meaning commercial exploitation, of polymetallic nodules has taken place, either in the CCZ or elsewhere. The ISA is currently developing exploitation regulations for deep-sea mining, for which environmental regulations will include governing waste discharge.^14,15^ In June 2021, the “two-year rule” at the ISA was triggered by the Republic of Nauru to complete exploitation regulations by July 2023. This date has now passed, but how that provision will be interpreted remains to be seen.^16^ Relevant scientific studies in the near term will be integral to the development of these regulations.

Environmental research to date has focused mostly on the impacts of nodule removal on the benthic environment and assessment of the resilience of benthic fauna and microbial ecosystems in the mining zones.^17−22^ In proposed mining operation workplans to date, the waste from shipboard dewatering of polymetallic nodules is delivered back to the water column.^23^ Importantly, the depth of waste discharge during potential future deep-sea mining operations, which could contain both crushed Mn nodules and deep-sea sediments, remains an open and controversial question. Naturally occurring manganese oxides are reductively dissolved in the ODZs of the Pacific Ocean, as evidenced by observations of low particulate Mn (<0.01 nM), high dissolved Mn (>1 nM) concentrations,^24−28^ and measurements of trace element redox couples.^29,30^ Based on these studies, we hypothesize that discharge of waste mining slurry containing crushed Mn nodule particles into an ODZ at mesopelagic depths will lead to reductive dissolution of discharged Mn oxides and the associated mobilization of heavy metals originally sorbed and incorporated into the Mn mineral phase of the nodules. This potential flux of reductively mobilized dissolved metals into ODZs is largely overlooked in the current literature of the environmental impacts of deep-sea mining,^14,31^ and the magnitude of these fluxes is central to the question of which depth dewatered mining waste should be discharged to the ocean.

In this study, we conducted incubation experiments to evaluate the potential mobilization of heavy metals from the reductive dissolution of Mn nodules during mining waste discharge into the ODZ overlying the CCZ. Trace metal clean sampling and analytical procedures enabled us to detect nanomolar concentration changes of dissolved trace metals associated with Mn nodule reduction and estimate the accumulation rates of Mn and other trace metals in the dissolved phase. This work offers one of the first geochemical perspectives of mining waste discharge in the mesopelagic ocean, evaluates a potential budget of metal release from the discharge plume, and explores the fate of these metals within the ODZ (dilution, sinking, complexation, etc.) to inform decisions related to future mining discharge operations.

## Experimental/Methods

### Mn Nodule Location and Characterization

Mn nodules used for the incubation experiment were collected from the Deep Ocean Mining Environmental Study (DOMES) Site A,^33^ at 8°27′ N and 150°47′ W, within the CCZ in the Equatorial North Pacific Ocean (Figure 1). Mn nodules were preserved at 4 °C since collection. Before the experiment, nodules with a length of ∼5 cm were crushed into microsized particles using a grinder in the laboratory to resemble the sediment slurry in the mining waste discharge. Crushed Mn nodules were characterized by the Brunauer–Emmett–Teller method^34^ (Micromeritics, Gemini VII) for surface area based on nitrogen adsorption isotherm measurements. X-ray diffraction was performed using an X’Pert3 powder diffractometer by Panalytical, scanning from 5° to 70° 2θ using a Cu Kα source. To distinguish among 10 Å manganates, samples were heated at 105 °C for 48 h and the run was repeated.

### Experimental Setup

To ensure anoxic conditions throughout the incubation, we used 125 mL Wheaton glass serum bottles, crimp seals with 3.25 mm thick polytetrafluoroethylene (PTFE)/silicone septa, and luer-lok syringes with Hamilton stainless steel needles (point style 2). Glass bottles were leached with 10% (v/v) reagent grade hydrochloric acid for 1 week, rinsed thoroughly with Milli-Q (MQ) water, and dried in a laminar flow bench before use. Seawater used for the incubation experiment was collected with a trace-metal-clean carousel using Go-Flo bottles (General Oceanics) at 200 m within the ODZ at Station 22 (9.2° N, 152.0° W) during the U.S. GEOTRACES GP15 cruise in 2018 (Figure 1), filtered using a 0.2 μm AcroPak-200 poly(ether sulfone) filter capsule (Pall), and stored in an acid-leached low-density polyethylene carboy.

At Day 0 of the experiment, 1.7 mg of crushed Mn nodule, equivalent to 9.9 μmol of Mn assuming 32.1 wt % of Mn, and 6.4 mg of trace metal grade sodium acetate were added to leached glass bottles before pouring ∼155 g of ODZ filtered seawater, resulting in approximately 64 μM of Mn and 500 μM of acetate (Figure S1). It has been shown that natural abiotic Mn oxide reduction is facilitated by labile dissolved organic carbon (DOC), which we represent as acetate, the reactant in the MnO_2_ reduction reaction.^35,36^ Two bottles with the same experimental setup were used for each group. All bottles were then immediately sealed with PTFE/silicone septa with aluminum caps and purged with a 900 ppm of CO_2_ and N_2_ gas mixture for 1 h to remove oxygen in the bottle while keeping pH relatively unchanged. The pH increased from 7.6 before purging to 7.9 after purging. Bottles were rotated at 170 rpm during the whole duration of the experiment using either a Boekel Wrist-O-Matic shaker (22 °C) or an IncuShaker Mini incubated shaker by Benchmark Scientific (42 °C) to ensure that the flow regime was fully turbulent.

### Sampling and Analytical Methods

Acid-leached luer-lok syringes with stainless steel needles were used to sample seawater at eight time points (Day 0, 1, 4, 8, 15, 22, 29, and 74) in a portable laminar flow hood (Sentry Air Systems) (Figure S1). Needles were rinsed with MQ water between different treatments and changed at different MnO_2_ concentrations. A new needle was used for each time point. About 2 mL of seawater was sampled each day and filtered through 0.2 μm acid-leached Whatman PTFE syringe filters for analysis of dissolved trace metals and nutrients. Dissolved trace metal samples were diluted ∼30 times with MQ water to 60 mL and promptly acidified to pH < 2 using quartz-distilled hydrochloric acid (HCl). After about 9 months of acidification, samples were analyzed for dissolved trace metals (Cd, lCo, Cu, Fe, Mn, Ni, Pb, Zn) at Texas A&M University using a preconcentration method on a SeaFAST-pico system (ESI, Omaha, NE) following a method modified from Lagerström et al.^37^ in Jensen et al.^38^ Preconcentrated trace metals were then analyzed on a Thermo Element XR high-resolution inductively coupled plasma mass spectrometer (HR-ICP-MS) at the R. Ken Williams Radiogenic Facility at Texas A&M University. Dissolved labile Co (dlCo) refers to weakly complexed and inorganic Co that is reactive to the Nobias PA1 resin used in the metal preconcentration. Nitrate and nitrite (NO_*x*_) were measured using a Lachat QuikChem 8000 Flow Injection Analyzer at the University of California Santa Cruz Marine Analytical Laboratory. The pH for all treatments was measured at room temperature at the end of incubation at Day 74 using a Thermo Scientific pH Benchtop meter. The pH ranged from 7.8 to 8.0 and was similar to the pH of ODZ seawater after purging measured at Day 0 (pH = 7.9), indicating minor pH changes during the incubation.

## Results and Discussion

In our experimental design (Table S1; Figure S1), we tested for the reduction of crushed Mn nodules (hereafter MnO_2_) and release of associated metals in the presence and absence of an added labile DOC source (acetate) and at two incubation temperatures, for a total of six groups: 64 μM MnO_2_ at room temperature (22 °C) (Group 1, G1: +Mn–Ac–T) and at 42 °C (Group 2, G2: +Mn–Ac+T); 64 μM MnO_2_ + 500 μM acetate at room temperature (Group 3, G3: +Mn+Ac–T) and at 42 °C (Group 4, G4: +Mn+Ac+T); and 500 μM acetate only at room temperature (Group 5, G5: –Mn+Ac–T) and at 42 °C (Group 6, G6: –Mn+Ac+T) (no MnO_2_ controls).

### Characterization of CCZ Mn Nodules Used for the Incubation

The specific surface area of the Mn nodules was 153.3 ± 0.7 m^2^ g^–1^. This value is lower than the surface area of biogenic Mn oxides produced by the bacterium *Leptothrix discophora* (224 m^2^ g^–1^)^39^ but comparable to previously reported specific surface area measurements of CCZ Mn nodules; ∼120 m^2^ g^–1.^^40^ Crushed CCZ nodules in this study contain 32.1 wt % of Mn, 9.3 wt % of Fe, 1.1 × 10^–3^ wt % of cadmium (Cd), 0.3 wt % of Co, 1.1 wt % of Cu, 1.5 wt % of Ni, and 6.5 × 10^–2^ wt % of lead (Pb), which are consistent with compiled major and minor chemical compositions of CCZ nodules.^13,41,42^ X-ray diffraction (XRD) results show that the starting material consisted mainly of 10 Å turbostratic phyllomanganates. Minor quartz detrital material is also apparent (Figure S2).

### Changes of Dissolved Trace Metals with Time during the Incubation

**Figure 2:**
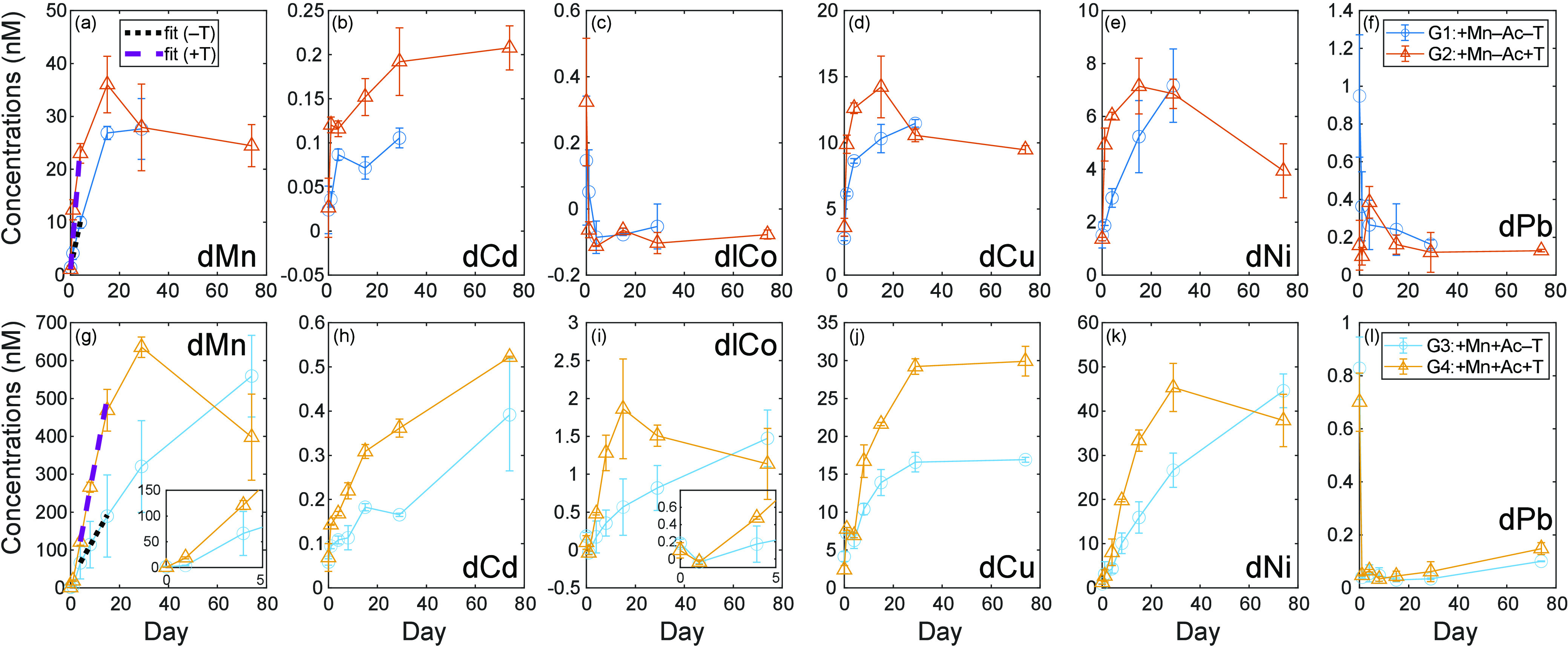
Dissolved trace metal concentrations (unit: nM) change with time during the incubation. (a–f) Trace metal concentrations (Mn, Cd, Co, Cu, Ni, Pb) in +MnO_2_ only groups with no acetate at 22 °C (G1: +Mn–Ac–T) and at 42 °C (G2: +Mn–Ac+T); (g–l): trace metal concentrations (Mn, Cd, Co, Cu, Ni, Pb) in +MnO_2_+acetate groups (G3: +Mn+Ac–T; G4: +Mn+Ac+T). Different groups (G1, G2, G3, or G4) are illustrated with different colored lines. Note that concentration scales change for each element between the two treatment groupings, the top and bottom rows. Error bars are standard deviations from duplicates. Weighted Model I regression is used to fit dissolved Mn concentrations during Days 0–4 for G1 and G2 and during Days 4–15 for G3 and G4, visualized as thick black and purple dashed lines, respectively. Insets in (g, i) are the expanded plots in the first 2 days of incubation for dissolved Mn and labile Co.

In groups G1–G4 with added MnO_2_, dissolved Mn (dMn) concentrations increased with time, at first linearly, in support of the method of initial rates most similar to open system conditions recorded for MnO_2_ reductive dissolution rates in previous studies.^43^ The increase was larger at higher temperature (G2 or G4) and in groups with higher acetate (G3 or G4) (Figure 2). The change of dMn from Day 0 to Day 1, however, was similar for groups with or without acetate: at 22 °C, the increase of dMn concentrations from Day 0 to 1 was 2.5 nM in the presence of acetate and 2.7 nM without acetate; at 42 °C, dMn increased by 18.4 nM and 11.2 nM with and without acetate, respectively. Given the similar and small increase in dMn between Days 0 and 1 regardless of acetate presence, we hypothesize that the nonreductive dissolution of Mn oxides or desorption of Mn^2+^ dominates in the first day (Figure 2).

We also monitored a competing electron acceptor to MnO_2_, nitrate plus nitrite (NO_*x*_), since the reduction of nitrate coupled to the oxidation of organic matter has similar Gibbs free energy to the reduction of MnO_2_,^44^ and nitrate is of much higher concentrations in ODZs than particulate Mn.^27,45^ The relatively unchanged NO_*x*_ concentrations from all groups at Days 0 and 1 further support the nonreductive release of dMn at the beginning of the incubation (Supporting Information Text & Figure S3). After Day 1, NO_*x*_ concentrations were quickly depleted in the presence of acetate (G4 or G3: +Mn+Ac±T) but decreased more slowly in the absence of acetate (G2 or G1: +Mn–Ac±T), suggesting that acetate was used as the electron donor in the reduction of NO_*x*_ (Figure S3). Similarly, the much higher rate of dMn accumulation in the presence of acetate after Day 1 suggests that acetate also acted as the electron donor in the reduction of MnO_2_. The slower accumulation of dMn in the absence of acetate (G2 or G1) after Day 1 suggests nonreductive dissolution or desorption processes occurring throughout the incubation and/or slower rates of reductive dissolution using the more recalcitrant background DOC as the electron donor. For simplicity, we will refer to processes in groups G2 or G1 (+Mn–Ac±T) as nonreductive.

Dissolved Mn^2+^ is known to strongly adsorb onto Mn oxide surfaces,^46^ but an overall net accumulation of dMn in all groups during the beginning of the incubation indicates the dominance of Mn dissolution and excess of desorption over adsorption. Concentrations of dMn then plateaued and decreased toward the end of the incubation, which can be explained by a gradual dominance of sorption of dissolved Mn^2+^ onto remaining Mn nodules in the closed system of our experimental setup. Secondary adsorption and related processes are less important in an open system such as the ocean where dissolved Mn^2+^ cannot easily accumulate.^47^

Similar to dMn, dissolved Cd (dCd), dissolved Cu (dCu), and dissolved Ni (dNi) concentrations increased with time in all groups (Figure 2) and were enhanced at higher temperatures (G4 or G2) and with the addition of acetate (G4 or G3). Dissolved labile Co (dlCo) decreased with time in the absence of acetate (G2 or G1: +Mn–Ac±T), possibly as a result of readsorption of released dlCo back onto remaining Mn nodule particles due to its high affinity for Mn oxides.^48−50^ A similar decrease in dlCo, about 0.1–0.3 nM, occurred initially between Day 0 and Day 1 in the presence of acetate (G4 or G3: +Mn+Ac±T), when nonreductive release of Mn dominated, but switched to net dlCo ingrowth after Day 1 once reductive dissolution of Mn nodules increased. Dissolved Zn and Fe concentrations are not discussed due to potential contamination issues (Supporting Information Text and Figure S4).

Dissolved lead (dPb) is also known to strongly scavenge onto Mn oxides.^39,51,52^ Unlike that of dlCo, the accelerated accumulation of dMn caused by the addition of acetate did not lead to a similar accumulation of dPb. Dissolved Pb concentrations decreased with time in all groups, implying that readsorption of dPb was faster than its release from any reductive dissolution of Mn nodules. The contrasting behavior between dlCo and dPb observed in G3 and G4 (+Mn+Ac) is consistent with laboratory reductive dissolution experiments using soil Mn nodules^53^ and with studies that show a higher readsorption rate of dPb onto Mn oxides than dlCo.^51,54−56^ Overall, the extent of dissolved trace metal accumulation in the incubation bottles was Mn > Cu > Ni > Cd > Co ≈ Pb in the absence of acetate (G2 or G1: +Mn–Ac±T) and Mn > Ni > Cu > Co > Cd > Pb in the presence of acetate (G4 or G3: +Mn+Ac±T).

### Rates and Metal Accumulation from Nodule Reduction during Incubation

Reductive dissolution of Mn oxides by organics under anoxic conditions has previously been demonstrated to be a surface- rather than transport-controlled reaction.^43,57,58^ We quantify dMn release from Mn nodules by calculating the slopes of the initial dMn concentration increase with time (Figure 2). Similar methods have been used to characterize Mn reduction rates under anoxic conditions using either synthesized Mn oxides^43,59^ or marine sediments.^60,61^ As expected, higher concentrations of dissolved Mn^2+^ accumulated from reductive dissolution of Mn nodules in G3 and G4 (+Mn+Ac) than from nonreductive dissolution processes in G1 and G2 (+Mn–Ac). Initial dissolved Mn accumulation rates from the linear (“open system”) portion at the beginning of the incubation (Day 0–4 for G1 and G2; Day 4–15 for G3 and G4) were 2.1 ± 0.3 nM day^–1^ for G1 (+Mn–Ac–T), 5.8 ± 0.4 nM day^–1^ for G2 (+Mn–Ac+T), 11.4 ± 9.9 nM day^–1^ for G3 (+Mn+Ac–T), and 34.5 ± 3.3 nM day^–1^ for G4 (+Mn+Ac+T) (Figure 2).

**Figure 3:**
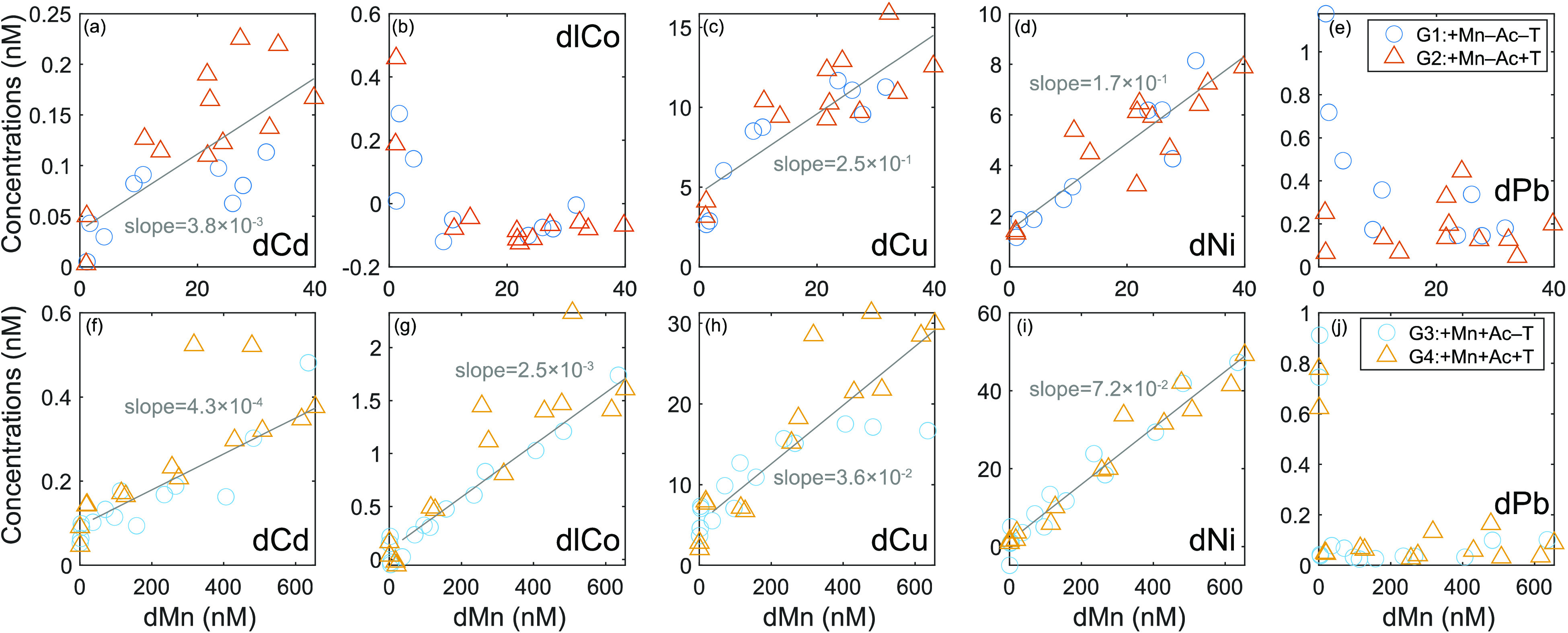
Relationships between dissolved trace metals and dissolved Mn (unit: nM). (a–e) +Mn–Ac groups (G1 and G2); (f–j) +Mn+Ac groups (G3 and G4); (a, f) dissolved Cd (dCd); (b, g) dissolved labile Co (dlCo); (c, h) dissolved Cu (dCu); (d, i) dissolved Ni (dNi); (e, j) dissolved Pb (dPb). Robust regression is used in the fit to minimize the effect of outliers on the linear relationships. Note that only data after Day 1 in G3 and G4 (+Mn+Ac) are included in the regression, since different processes likely occur before and after Day 1. Only regression fits with *p* values smaller than 0.05 are shown. Again, note that concentration scales change for each element between the two treatment groupings.

The trace metal release in different groups, as defined by the ratios between the accumulation of dissolved trace metals and dMn (dTMs/dMn), was calculated to quantitatively evaluate which metals were more/less accumulated than one might expect based on congruent (stoichiometric) nodule dissolution (Figure 3; Table S2). In the absence of acetate (G1 and G2: +Mn–Ac), estimated dTM/dMn ratios and their standard errors were (3.8 ± 0.9) × 10^–3^ mol Cd/mol Mn, (2.5 ± 0.4) × 10^–1^ mol Cu/mol Mn, and (1.7 ± 0.2) × 10^–1^ mol Ni/mol Mn. In the presence of acetate (G3 and G4: +Mn+Ac), estimated dTM/dMn ratios were (4.3 ± 0.6) × 10^–4^ mol Cd/mol Mn, (2.5 ± 0.2) × 10^–3^ mol lCo/mol Mn, (3.6 ± 0.4) × 10^–2^ mol Cu/mol Mn, and (7.2 ± 0.5) × 10^–2^ mol Ni/mol Mn.

**Figure 4:**
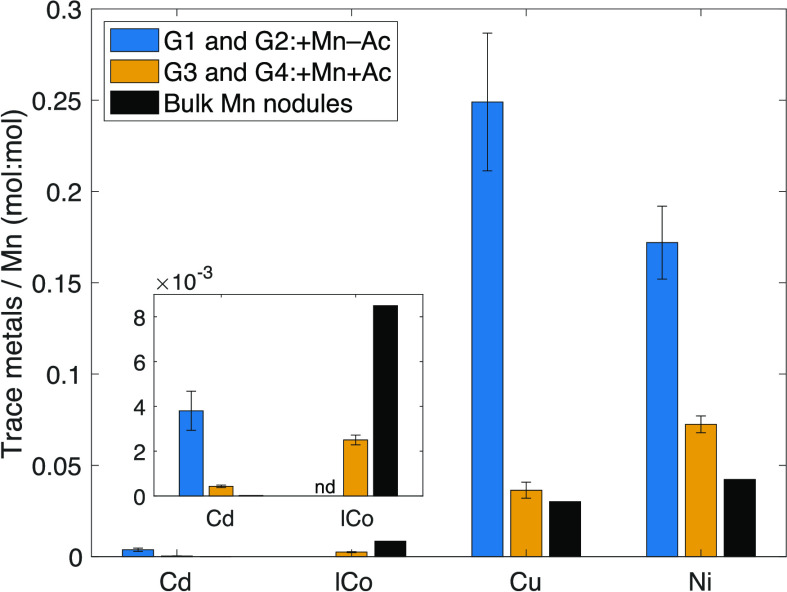
Bar plots of the ratios between trace metals and Mn (unit: mol/mol) of dissolved phases in different incubation groups (dTMs/dMn) compared with concentrations measured in solid bulk nodules used for the incubation (TMs/Mn)_nodule_. Error bars are standard errors of the dTMs/dMn slopes in G1 and G2 (+Mn–Ac) and in G3 and G4 (+Mn+Ac). Inset is the expanded bar plot of Cd and Co. The ratio of dlCo/dMn in G1 and G2 is not determined due to strong readsorption of dlCo onto Mn oxide surfaces.

The bulk molar ratios of crushed Mn nodules (unit: mol/mol) used for the incubation were 1.7 × 10^–5^ for (Cd/Mn)_nodule_, 8.5 × 10^–3^ for (Co/Mn)_nodule_, 3.0 × 10^–2^ for (Cu/Mn)_nodule_, and 4.2 × 10^–2^ for (Ni/Mn)_nodule_ (Table S2). These trace metal-to-Mn bulk molar ratios in nodules (TMs/Mn)_nodule_ were similar to the dTMs/dMn ratios in solution in G3 and G4 (+Mn+Ac) and were about one magnitude lower than the dTMs/dMn ratios in G1 and G2 (+Mn–Ac) (Figure 4). The order of magnitude higher dTMs/dMn ratios in the nonreductive G1 and G2 (+Mn–Ac) experiments show that some dTMs nonreductively dissolve or desorb from the MnO_2_ surface upon meeting seawater, which is relevant to mining waste discharge at any depth (not just ODZs), as it is independent of reductive conditions. In contrast, the overall extent and similarity between dTMs/dMn ratios and bulk Mn nodule composition in the presence of acetate (G3 and G4), especially dCu/dMn and dNi/dMn, confirm that a source of acetate appears to facilitate the reduction of the Mn nodule under anoxic conditions and release trace metals that are structurally incorporated in the MnO_2_ octahedral layers.

The relative abundance of Cu and Ni released is affected by the presence or absence of acetate: more Ni than Cu was released during reductive dissolution in G3 and G4 (+Mn+Ac), but the opposite was true during nonreductive dissolution in G1 and G2 (+Mn–Ac). This may be due to the structural incorporation into phyllomanganate layers being more favorable for Ni (∼10–45% incorporated into layer vacancy sites)^62,63^ than Cu (∼0–20% occupying vacancy sites)^64,65^ at pH 7–8, whereas the desorption from the interlayer between MnO_6_ octahedral layers and layer edge sites is likely to favor Cu over Ni.

The dCd/dMn ratio in the presence of acetate (G3 and G4: +Mn+Ac) was ∼25 times higher than (Cd/Mn)_nodule_ of 1.7 × 10^–5^ for bulk Mn nodules, whereas the dlCo/dMn ratio was ∼3.5 times lower than (Co/Mn)_nodule_ of 8.5 × 10^–3^ (Figure 4; Table S2). Differences between dTMs/dMn measured in the incubation seawater in the presence of acetate and the (TMs/Mn)_nodule_ of bulk Mn nodules suggest that reductive dissolution is not the only important process during the incubation. Readsorption of released dTMs back onto remaining Mn nodules is also relevant, and its relative magnitude varies between different trace metals (Figure S5): a higher readsorption than that of dMn leads to a smaller accumulated dTMs/dMn than (TMs/Mn)_nodule_ of bulk Mn nodules assuming congruent dissolution. For example, the dlCo/dMn ratio is smaller than the bulk (Co/Mn)_nodule_ ratio because dlCo readsorbs more than dMn to the Mn nodule surface, but the dCd/dMn ratio is larger than its nodule stoichiometry because there is less readsorption for dCd. Therefore, our incubation experiment demonstrates that the sorption affinity of dissolved metals onto Mn nodules follows a general order of Cd < Ni < Cu ≈ Mn < Co < Pb, which is consistent with previous sorption experiments^54,66−68^ and the thermodynamic stability of surface complexation.^69^

### Consideration of Relevance to Dewatering Operations

There are three aspects to consider, temperature, MnO_2_ concentrations, and microbial abundances, before extrapolating our incubation experiments to a potential mining waste plume scenario.

First, the temperature effect of the reductive dissolution of Mn oxides by various organic compounds has previously been modeled using the Arrhenius equation between the temperature range of 5 to 40 °C.^57,70−72^ We can estimate Mn reduction rates at the temperature of ODZ seawater at 200 m overlying the CCZ (approximately 11 °C)^73^ based on rates from our incubation experiments using the Arrhenius equation, with potential error quantified for our results and also taking into account previous analyses for similar systems (Supporting Information Text). We model the kinetics of MnO_2_ reduction as a second-order reaction^74^ with respect to concentrations of MnO_2_ and DOC for groups G3 and G4 (+Mn+Ac) where MnO_2_ reduction occurred.
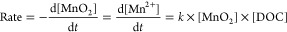
1where  and  are the change of Mn oxides and dissolved Mn concentrations with time, respectively, *k* is the second-order rate constant (unit: L mol^–1^ day^–1^), and [MnO_2_] and [DOC] are concentrations of MnO_2_ and DOC, respectively. The incubation of Mn nodules is conducted with excess concentrations of DOC (acetate + background DOC ≈ 550 μM) and Mn nodules ([MnO_2_] = 64 μM). The rate of Mn oxide reduction, therefore, can be treated as pseudozero order with respect to the initial concentration of Mn oxides and DOC in the bottle as follows:
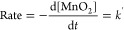
2where *k*’ is the pseudozero-order rate constant (unit: mol L^–1^ day^–1^). The apparent activation energy *E*_a_ of the reaction can be estimated using two temperatures (22 and 42 °C) based on the Arrhenius equation:
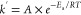
3
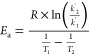
4where *A* is a pre-exponential factor (unit: mol L^–1^ s^–1^), *E*_a_ is the activation energy (unit: J mol^–1^), *R* is the gas constant (8.314 J mol^–1^ K^–1^), *T* is the temperature (unit: K), and subscripts 1 and 2 represent the parameter (rate constant or temperature) at two different temperatures.

Using dMn accumulation rates calculated from our incubations in the presence of acetate (G3: *k*’ = 11.4 nM day^–1^ at 22 °C; G4: *k*’ = 34.5 nM day^–1^ at 42 °C), the apparent activation energy *E*_a_ is estimated as 42.8 kJ mol^–1^. This *E*_a_ falls within the range of 17.4–60 kJ mol^–1^ found in previous kinetic studies of the reductive dissolution of Mn oxides,^57,70−72^ consistent with a surface-controlled reaction.^47^ We can then derive a dMn accumulation rate of 5.8 nM day^–1^ for an ODZ temperature of 11 °C as the pseudozero-order rate constant *k*’ (eq 4).

Second, we compared concentrations of MnO_2_ used in our incubation to the dewatering waste plume to confirm that they are comparable after accounting for turbulent mixing upon waste discharge. To estimate dewatering plume composition, we utilize the composition of a sediment plume created by mobilization of CCZ sediments from the nodule miner with and without added crushed Mn nodule material. We use the density for a commercial sediment plume in the CCZ of 8 g L^–1^.^75^ The lower bound of particulate Mn concentrations in the waste plume is 24 mg L^–1^ (436.4 μM), which assumes crushed Mn nodules are absent from the waste plume and the plume is entirely composed of surface CCZ sediments without nodules that are characterized by about 0.3 wt % of leachable particulate Mn.^76^ As an upper bound, we assume that the discharged waste sediment consists of entirely crushed Mn nodules that are about 30 wt % Mn, equivalent to 2400 mg L^–1^ or 43.6 mM of particulate Mn. A recent field study discharging CCZ sediments into the subsurface Northeast Pacific Ocean demonstrated that the descending waste plume is diluted 100–1000 times upon discharge by ambient seawater via strong turbulent mixing as the *dynamic* plume and then laterally advected by ocean currents as the *ambient* plume.^75^ Such dilution decreases the particulate Mn concentration and sets the initial particulate Mn concentrations at 0.4–436.4 μM in the *ambient* plume. Therefore, the MnO_2_ concentration used in our incubation experiment, 64 μM particulate Mn, falls within the range of the initial MnO_2_ concentration of the *ambient* waste plume, which suggests that both zero-order kinetics and the relative rates that we found may be applicable.

Third, it remains unclear how relevant our estimated abiotic dMn accumulation rates are compared to *in situ* Mn reduction rates within the ODZ that are mediated by microbial processes. To the best of our knowledge, there have not been any direct field measurements of MnO_2_ reduction or dMn accumulation rates within any ODZs in the water column. To estimate in situ MnO_2_ reduction rates, we assume that previous observations from the eastern tropical Pacific that show a decrease in Mn oxide concentrations in ODZs are because of reductive dissolution.^24,27,28^ We can use observations of the decrease of Mn oxides with depth from the U.S. GEOTRACES Eastern Pacific Zonal Transect (GP16) in the Peruvian ODZ^77^ to estimate a pseudo-first-order rate constant of Mn reduction of (1.6–8.4) × 10^–3^ day^–1^, using the sinking time scale for a Mn oxide particle according to Stokes’ Law^78^ (Supporting Information Text). Using this *in situ* rate constant for Mn reduction from the GP16 cruise to approximate the *ambient* plume under anoxic conditions in the CCZ, we estimate MnO_2_ reduction rates within the *ambient* plume as 0.7–3686 nM day^–1^ given MnO_2_ concentrations of 0.4–436 μM. The dMn accumulation rate of 5.8 nM day^–1^ at 11 °C derived from our abiotic Mn nodule reduction incubation experiments is at the low end but comparable to our calculated *in situ* MnO_2_ reduction rates that might occur if dewatered mining waste was discharged into ODZs (0.7–3686 nM day^–1^). Note that the dMn accumulation rates in our incubations include strong readsorption of Mn^2+^ onto Mn oxide surfaces^46,60^ and can be up to 10 times lower than MnO_2_ reduction rates as shown in anoxic incubations of marine sediments in the East Sea^61^ and the Norwegian Trough.^60^ If we consider the effects of strong readsorption onto Mn oxides in the waste plume, the dMn accumulation rate within the initial *ambient* plume would be 0.07–368.6 nM day^–1^. Thus, the dMn accumulation rate from our incubation may serve as a representative rate, which is very similar to the estimated geometric mean of 5.0 nM day^–1^ within the projected waste plume should it be discharged to the ODZ.

### Estimations of Trace Metal Budgets within the Waste Plume

**Figure 5:**
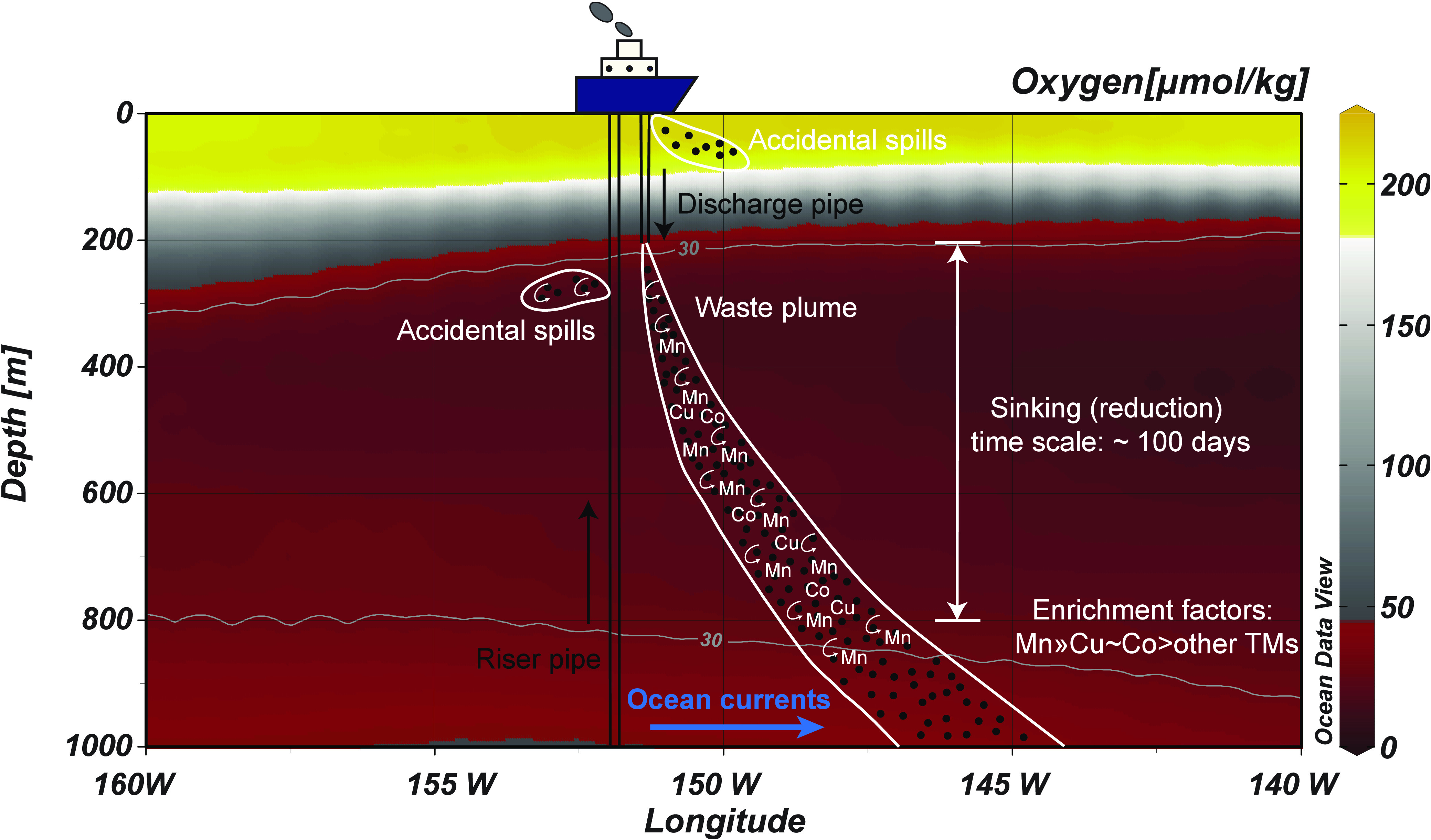
Schematic of the trace metal inputs in a potential deep-sea mining dewatering waste plume and accidental spills from both the riser pipe and the mining platform. The section plot is dissolved oxygen concentrations (unit: μmol kg^–1^) along 12° N. Dissolved oxygen data are retrieved from the World Ocean Atlas 2018 annual climatology,^32^ and the gray contours are oxygen concentrations of 30 μmol kg^–1^. The most enriched trace metals, in comparison to the background seawater concentrations, that are released from the reductive dissolution of Mn nodules are shown as white, curved arrows.

We hypothesize that the trace metal compositions (dTMs/dMn) observed during our experiments are applicable to potential future waste plumes, which would include direct dewatered waste discharge as well as any accidental spills from either the riser pipe or the ship into surface waters, which would settle through the ODZs (Figure 5). Insights into trace metal budgets during mining dewatering operations, therefore, can be gained by extrapolating our experimental results to a waste discharge scenario. We use the dMn accumulation rate from our incubation, representative of rates within the initial *ambient* plume, to estimate the overall budget of dissolved Mn and other trace metals that may result from discharging a mining waste plume within the ODZ.

Our incubation results suggest that Pb likely readsorbs onto remaining Mn surfaces after reductive dissolution of Mn oxides, whereas other trace metals (Cd, Co, Cu, and Ni) are released into the water column by both nonreductive but much more so by reductive dissolution processes (Figure 2). Trace metal accumulation rates in a hypothetical waste plume are estimated using the dMn accumulation rate (5.8 nM day^–1^) and trace metal composition during the reductive dissolution of Mn nodules (Figure 3f–i). Estimated accumulation rates in the ODZ seawater, therefore, are 2.5 × 10^–3^ nM day^–1^ for Cd, 1.4 × 10^–2^ nM day^–1^ for Co, 2.1 × 10^–1^ nM day^–1^ for Cu, and 4.2 × 10^–1^ nM day^–1^ for Ni.

How significant are these accumulation rates of trace metals in ODZ seawater? Are released trace metal concentrations from the waste plume much higher than the background level? The upper bound of concentrations of trace metals in seawater that encounter the waste plume occurs in the absence of further dilution within the *ambient* plume. During the ∼100 days of sinking required to pass through the 600 m-thick ODZ (Supporting Information Text), the amount of dMn resulting from a single mining waste plume is estimated as 1.0 nM (55.0 ng L^–1^ of Mn) distributed uniformly across the ODZ. The most extreme case of continuous waste discharge, in which a plume of MnO_2_ particles is reductively reduced across the entire thickness of the ODZ, would lead to 580 nM dMn (equivalent to 31.9 μg L^–1^ of Mn) released during the sinking time scale under steady state. The estimated upper limit of plume-sourced dMn (1–580 nM) exceeds background dMn concentrations of 0.5–1.0 nM in the ODZ of the Eastern Tropical Pacific Ocean close to the CCZ by 2 orders of magnitude.^79^ Likewise, the input of other dTMs into the mesopelagic ocean of the CCZ would be 4.3 × 10^–4^ to 2.5 × 10^–1^ nM for Cd (4.8 × 10^–2^ to 2.8 × 10^1^ ng L^–1^), 2.5 × 10^–3^ to 1.5 × 10^0^ nM for Co (1.5 × 10^–1^ to 8.6 × 10^1^ ng L^–1^), 3.6 × 10^–2^ to 2.1 × 10^1^ nM for Cu (2.3 × 10^0^ to 1.4 × 10^3^ ng L^–1^), and 7.3 × 10^–2^ to 4.2 × 10^1^ nM for Ni (4.3 × 10^0^ to 2.5 × 10^3^ ng L^–1^) (Table S3). The background concentrations of dCd, dlCo, dCu, and dNi at the mesopelagic depths of ODZ in the Eastern Tropical Pacific Ocean are about 0.1–1.1 nM, 0.03–0.1 nM, 0.6–1.6 nM, and 3–8 nM, respectively.^79^ These results suggest that Co or Cu could be the most enriched trace metal within the waste plume compared to the background seawater concentrations. Concentrations of dlCo, dCu, and dNi associated with Mn oxide reduction within the waste plume can be 0.02–15.0, 0.02–13.1, and 0.009–5.2 times the highest concentrations found in the mesopelagic, respectively, whereas dCd concentrations would be diluted by the mining waste discharge (Figure 5; Table S3). Metal release during nonreductive dissolution of MnO_2_, such as into water depths outside of the ODZ, would be lower and have a slightly different elemental profile (see data from G1 and G2 experiments).

The possible enrichment of dCu in the waste plume may be a concern given its strong toxicity to some phytoplankton.^80−82^ Cu is strongly complexed by organic ligands in the ocean,^83^ and the toxicity of Cu mostly manifests in its inorganic form. Paul et al.^84^ detected high concentrations of excess Cu-binding ligands (up to ∼200 nM) in bottom waters and deep-sea pore waters of CCZ sediments, and they argued that excess ligands could chelate some of the Cu through organic complexation and reduce its toxicity to benthic fauna during future mining operations. Concentrations of excess organic Cu-binding ligands within the ODZ are up to 2 nM in the Eastern Tropical South Pacific,^85^ which can only bind part of the Cu^2+^ we project to be released from the waste plume at mesopelagic depths. How the remainder of Cu (up to 18 nM) from the waste discharge plume would influence the mesopelagic ecosystem remains unclear. For context, 18 nM of dCu is higher than typical concentrations of Cu in the open ocean (maximum ∼4–5 nM near the bottom)^79^ but falls on the lower end of dCu concentrations in pelagic sediment pore waters in the Pacific.^84,86^ Two aspects of added Cu from the waste discharge at mesopelagic depths, beyond complexation by excess organic ligands, that could supplement the findings from our study are (1) The toxicity threshold of Cu for mesopelagic ecosystems (e.g., zooplankton and fishes) is unknown. Such thresholds vary between different plankton communities,^80,87^ and better baseline studies of Cu ecotoxicology are needed for mesopelagic communities. (2) The behavioral response of mesopelagic communities to added Cu is also unknown. Increased ligand production has been reported for both cyanobacteria and eukaryotic phytoplankton in response to the addition of high levels of Cu.^82,88^ It remains unclear whether mesopelagic microbial communities would have similar responses, and this also requires a future investigation.

This study provides a geochemical perspective of the influence of mining based on current proposals and offers a feasible metal release budget if the mining wastewater were to be discharged into the ODZ. Importantly, the trace metal budgets presented here are estimated using a dMn accumulation rate derived from incubation experiments. This rate is likely more representative of dMn accumulation rates in the early phase of the waste plume, before any further dilution occurs during plume advection (dilution factors up to 40,000–400,000),^75^ thereby serving as an upper bound for the waste discharge scenario. The estimated trace metal budget may also change due to its sensitivity to factors such as the choice of dMn accumulation rates and the concentrations and compositions of the sediments in the waste plume (Supporting Information Text). Future studies on in situ dMn accumulation rates during the transport of the waste plume would help better constrain trace metal budgets during mining dewatering operations.

## Conclusions

This work demonstrates the potential release of many heavy metals due to the reductive dissolution of Mn nodules if the deep-sea mining wastewater were to be discharged into the ODZ. As interest in Mn nodule deep-sea mining continues to increase, it is vital that we understand the influence of mining dewatering waste to trace metal biogeochemical cycling, especially for potentially toxic dissolved Cu, and its potential impact on the mesopelagic ecosystem. Based on our incubation results, we expect to see a slower release of metals due to nonreductive dissolution processes if released outside of the ODZ, which is critical information for choosing the depth of dewatered mining waste discharge. However, regardless of the discharge pipe depth, Mn nodule reductive dissolution remains important as a result of inevitable surface spills that will settle MnO_2_ particles through the ODZ layer. Overall, more work on the release of trace metals into oxygen-rich waters above and below the ODZs is needed in addition to studies of putative metal toxicity to mesopelagic communities in order to gauge ecological effects of these fluxes.

## Data Availability

The nutrients and trace metal concentration data that support the findings of this study are available in Zenodo (10.5281/zenodo.7776211).

## Supporting Information Available

Additional experimental methods, results, and sensitivity studies (PDF)
