# Supplementary material for: Metal Release from Manganese Nodules in Anoxic Seawater and Implications for Deep-Sea Mining Dewatering Operations
Source: ACS ES T Water. 2024 Jun 27;4(7):2957–67. doi: 10.1021/acsestwater.4c00166 (PMC11249780; doi:10.1021/acsestwater.4c00166)
Supplement: Supplementary file 1 — ew4c00166_si_001.pdf [file ew4c00166_si_001.pdf]

Supporting Information for

Metal release from manganese nodules in anoxic seawater and implications for deep-sea mining dewatering operations

Yang Xiang[1,†,], Janelle M. Steffen[2], Phoebe J. Lam[1], Amy Gartman[3,*], Kira Mizell[3], Jessica N. Fitzsimmons[2]*

[1]Department of Ocean Sciences, University of California, Santa Cruz, CA 95064, United States

[2]Department of Oceanography, Texas A&M University, College Station, TX 77840, United States

[3]U.S. Geological Survey, Pacific Coastal and Marine Science Center, Santa Cruz, CA 95060, United States

[*]Corresponding authors: Yang Xiang (yaxiang@ucsc.edu), Amy Gartman (agartman@usgs.gov)

[†]Present address: Cooperative Institute for Climate, Ocean, and Ecosystem Studies, University of Washington, Seattle, WA 98105, United States

Supporting Information Text

Changes of nitrate+nitrite with time during the incubation. In all groups, nitrate+nitrite (NO_3+NO_2 or NO_x) concentrations generally decreased with time (Fig. S3). The extent of NO_x decrease was strongly influenced by the addition of acetate, but also slightly by temperature and the presence of Mn oxides. With no added acetate, NO_x decreased slightly in the first 8 days of incubation in both G2 or G1 (+Mn–Ac±T), with the higher temperature in G2 leading to a more pronounced NO_x decrease of about 5 μM between Days 8 and 74. The trend of NO_x in groups with 500 μM added acetate (G3-G6) all displayed an abrupt decrease of ~20 μM NO_x after Day 1 and remained essentially zero. Concentrations of NO_x reached 0 at Day 8 in G4 (+Mn+Ac+T), whereas NO_x was completely depleted earlier at Day 4 in the other three groups (G3: +Mn+Ac–T; G5: –Mn+Ac–T; G6: –Mn+Ac+T) (Fig. S3b-c). Since there was no dependence on temperature in the absence of MnO_2 (Fig. S3c), we attribute the slower drawdown of NO_x in G4 (+Mn+Ac+T) (Fig. S3b) to the competing influence of Mn oxides, which has similar Gibbs free energy as NO_x to act as an electron acceptor in the anoxic oxidation of organic matter [1]. Additionally, despite the much faster decrease of NO_x in the presence of acetate after Day 1, there was little decrease in NO_x between Days 0 and 1 for all groups, suggesting that the reduction of NO_x did not start until after Day 1.

Changes of dissolved zinc and iron with time during the incubation. Dissolved trace metal data from the groups with added acetate but not MnO_2 (G6 or G5: –Mn+Ac±T) served as a control for identifying potential trace metal contamination from glass bottles, sampling, and from the acetate addition (Fig. S4). For example, dissolved zinc (Zn) concentrations increased rapidly and were characterized by similar or even higher

concentrations than dissolved Mn (Figure 2), which was likely caused by contamination from glass bottles. Likewise, contamination from glass bottles and/or stainless-steel needles used for sampling may explain the noisy dissolved iron (dFe) data. The low and similar magnitude of maximum dFe concentrations between groups with and without Mn nodules suggests that the reduction of Fe from nodules was not important in our incubation experiment, which is consistent with the absence of active Fe(III) reduction observed within the oxygen deficient zones (ODZs) in the Pacific Ocean [2-4].

Estimating Mn oxide reduction rates in ODZs during the U.S. GEOTRACES GP16 cruise. Surface Mn oxide concentrations, estimated as non-lithogenic particulate Mn, reach a maximum of 0.4 nM at 45 m and a minimum of 1.8×10^{-3} nM at 235 m at Station 1 (12.0˚S, 79.2˚W) during the U.S. GEOTRACES GP16 cruise in the Peruvian ODZ [5]. At Station 4 (12.0˚S, 77.8˚W), concentrations of Mn oxides decrease from 0.15 nM at 20 m to 4.0×10^{-4} nM at 300 m. We estimate the time scale of Mn reduction as the time for a Mn oxide particle to sink between the depths of the maximum and minimum Mn oxide concentrations according to Stokes' Law [6]. Assuming particle density as pure Mn oxides of 3.0 g cm^{-3}, seawater density of 1.027 g cm^{-3}, viscosity as 1.25×10^{-3} kg m^{-1} s^{-1}, and size of Mn oxide particles of 1–2 μm [7], we estimate a Stokes' sinking velocity of 0.1–0.3 m d^{-1}, leading to a reduction time scale at Station 1 as 640–2,559 days and at Station 4 as 943–3,771 days. The pseudo-first-order rate constant of Mn reduction at Stations 1 and 4 within the ODZ during the GP16 cruise is 1.6×10^{-3}–8.4×10^{-3} day^{-1} assuming excess of DOC concentrations (typical of 50 μM at ~200 m at Stations 1 and 4) [8].

Estimating residence time for crushed Mn nodules in the ODZ within the CCZ. The *ambient* plume is characterized by low particle concentrations and not expected to be

influenced by strong particle aggregation [9]. Crushed Mn nodules in the waste plume have been estimated to have a median size of 9–12 μm [9], too small to be easily recovered but still larger and thus faster sinking than the 1–2 μm size of naturally-occurring particulate Mn (pMn) [7]. Assuming a median size of 9–12 μm [9], a particle density of pure Mn oxides of 3.0 g cm^{-3}, seawater density of 1.027 g cm^{-3}, and viscosity of 1.37×10^{-3} kg m^{-1} s^{-1} for 11 ˚C seawater, we calculate a sinking velocity using Stokes' Law [6] of about 5.5–9.8 m d^{-1} for MnO_2 particles of this size in the waste plume. Such sinking rates are very similar to the 0.1 mm s^{-1} (8.6 m d^{-1}) previously calculated by Muñoz-Royo et al. [9]. It would take about 62 to 109 days to sink through the entirety of a 600 m-thick ODZ region.

Sensitivity tests of the estimated trace metal budgets. The trace metal budgets calculated in this study likely fall on the higher end of the metal budgets of a waste plume due to turbulent mixing during the *ambient* plume advection. The laterally transported *ambient* plume would be further diluted beyond the initial dilution factor of 100–1,000 at the *dynamic* plume stage. Muñoz-Royo et al. [9] modeled the extent of the plume for 90 days and used dilution factors of 40,000–400,000, demonstrating further dilution of more than 40 times during the transport of the *ambient* plume. This level of dilution would lead to similar or even lower concentrations of all measured trace metals (Mn, Cd, Co, Cu, and Ni) within the ambient plume than the background seawater concentrations.

Trace metal release rates and budgets are also sensitive to factors such as different dMn accumulation rates, and the concentrations and compositions of the sediment plume. If a potential lower (0.07 nM day^{-1}) or upper (368.6 nM day^{-1}) bound of dMn accumulation rate within the waste plume was used, dissolved trace metal accumulation rates could correspondingly decrease by 82.9 times or increase by 63.6 times. The upper bound

scenario leads to concentrations of all trace metals (Mn, Cd, Co, Cu, and Ni) far exceeding their background concentrations within the ODZ, whereas even Cu, Co, and Ni accumulation rates would be smaller than the background level in the lower bound scenario. Additionally, if sediment concentrations of the waste plume increased from 8 to 32 g L^{-1}, all trace metal accumulation rates would increase by a factor of four. In comparison, the trace metal budget is less sensitive to the choice of the apparent activation energy used in the calculation. If we use activation energies of 17.4 and 60 kJ mol^{-1}, the lower and upper bounds reported in the literature [10-13], dMn accumulation rates at 11 ˚C become 8.7 and 4.4 nM day^{-1}, respectively. This is equivalent to a relative change of 49.3% and –23.8% as compared to 5.8 nM day^{-1}, which is much smaller than other sources of uncertainties.

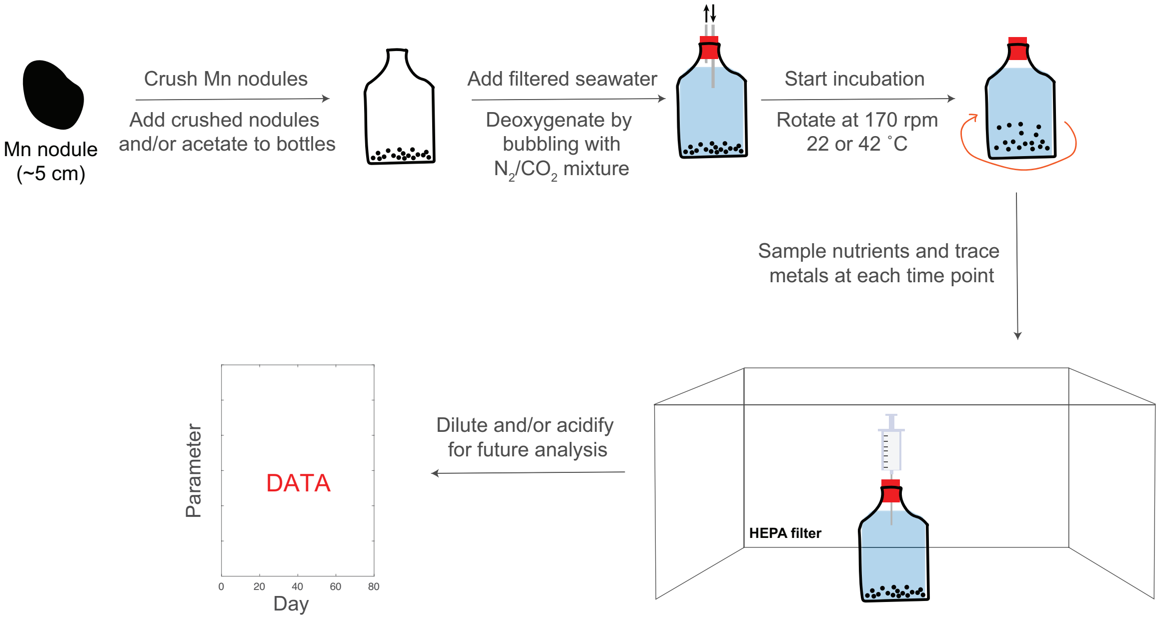

Fig. S1. Schematic of the experimental setup. Seawater at each time point was sampled using syringe filters in a portable laminar flow hood.

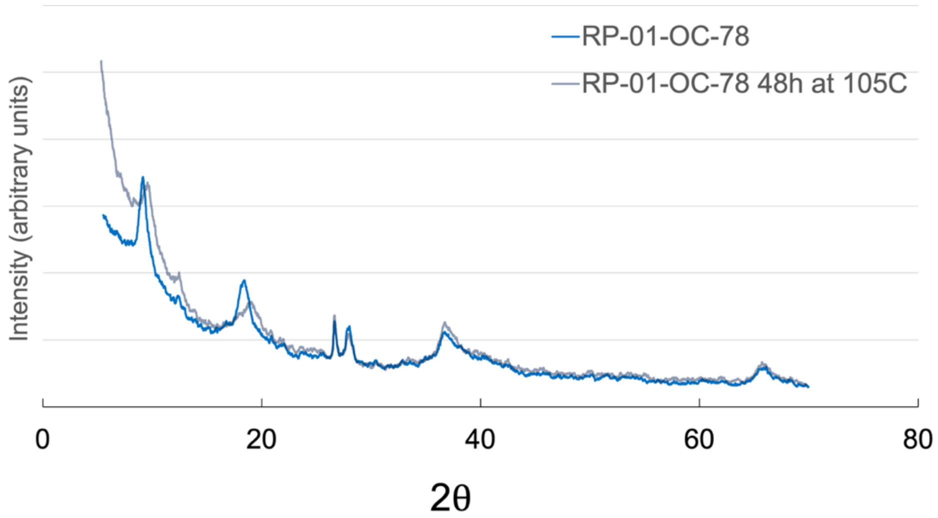

Fig. S2. X-ray diffraction (XRD) pattern of CCZ Mn nodules used for the incubation experiment. The blue line is the initial XRD pattern, and the grey line is the XRD pattern of nodules heated at 105 ˚C for 48 hours.

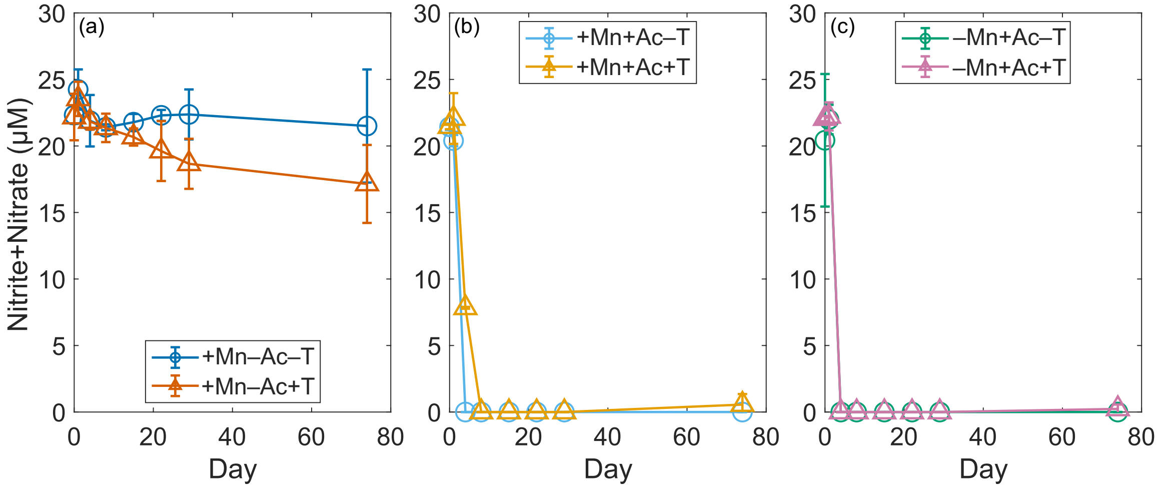

Fig. S3. Nitrate+nitrite (NO_x) concentrations (unit: μM) change with time during the incubation. (a) MnO_2 only groups (G1: +Mn–Ac–T; G2: +Mn–Ac+T); (b) MnO_2+acetate groups (G3: +Mn+Ac–T; G4: +Mn+Ac+T); (c) acetate only groups (G5: –Mn+Ac–T; G2: –Mn+Ac+T). Error bars are standard deviations from duplicates. Different groups are illustrated with different colored lines.

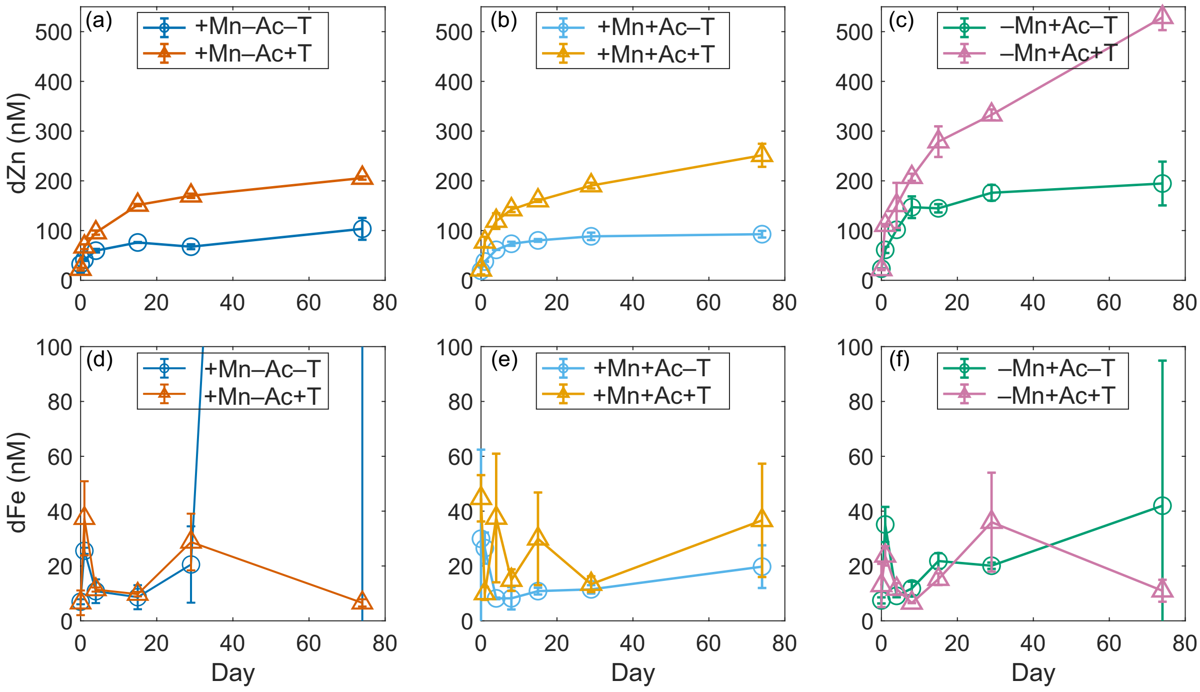

Fig. S4. Dissolved zinc (dZn) and iron (dFe) concentrations (unit: nM) change with time during the incubation. (a): dZn in MnO_2 only groups (G1: +Mn–Ac–T; G2: +Mn–Ac+T); (b): dZn in MnO_2+acetate groups (G3: +Mn+Ac–T; G4: +Mn+Ac+T); (c): dZn in acetate only groups (G5: –Mn+Ac–T; G2: –Mn+Ac+T); (d): dFe in MnO_2 only groups (G1 and G2); (e): dFe in MnO_2+Ac groups (G3 and G4); (f): dFe in acetate only groups (G5 and G6). Error bars are standard deviations from duplicates. Lower dZn concentrations in (a) and (b) than (c) indicate strong adsorption of dZn released from glass bottles by Mn nodules. Note that dFe concentration at Day 74 in G1 is 1154.5±1422.7 nM (mean±s.d.), much higher than the y-scale of the plot.

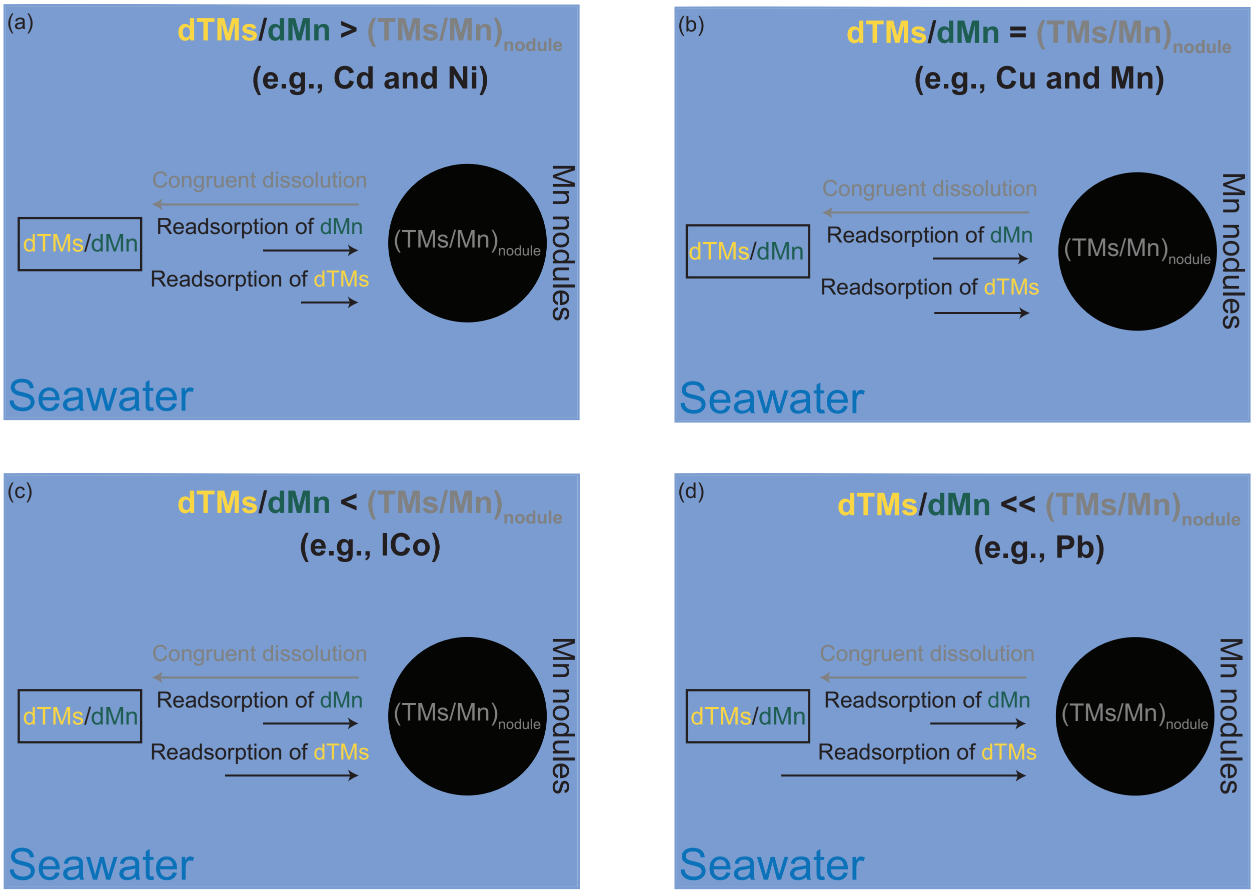

Fig. S5. Schematic of dissolution and readsorption for different trace metals during the reductive dissolution of Mn nodules in our incubation experiments. The stoichiometry of trace metal accumulation in seawater and the bulk Mn nodules is dTMs/dMn and $(TMs/Mn)_{nodule}$, respectively. The elements and arrow size are changing with each panel. The length of the arrows in each category reflects the relative magnitude of each process.

Table S1. Summary of six groups of the incubation experiment

	Added MnO_2 concentrations (μM)	Added acetate concentrations (μM)	Temperature (˚C)	Abbreviations
Group 1 (G1)	64	0	22	+Mn–Ac–T
Group 2 (G2)	64	0	42	+Mn–Ac+T
Group 3 (G3)	64	500	22	+Mn+Ac–T
Group 4 (G4)	64	500	42	+Mn+Ac+T
Group 5 (G5)	0	500	22	–Mn+Ac–T
Group 6 (G6)	0	500	42	–Mn+Ac+T

Table S2. Summary of ratios between trace metals and Mn (unit: mol:mol) in different groups

	Cd/Mn	Co/Mn	Cu/Mn	Ni/Mn
G1 and G2 (+Mn–Ac)	$3.8\pm0.9\times10^{-3}$	nd	$2.5\pm0.4\times10^{-1}$	$1.7\pm0.2\times10^{-1}$
G3 and G4 (+Mn+Ac)	$4.3\pm0.6\times10^{-4}$	$2.5\pm0.2\times10^{-3}$	$3.6\pm0.4\times10^{-2}$	$7.2\pm0.5\times10^{-2}$
Bulk Mn nodules	1.7×10^{-5}	8.5×10^{-3}	3.0×10^{-2}	4.2×10^{-2}

Table S3. Estimated dissolved trace metal concentrations in seawater within the CCZ and their enrichment factors

Trace metals	Concentrations in the CCZ (nM) [14]	Estimated concentrations from the waste plume (nM)	Enrichment factors*
Mn	0.5–1.0	1–580	1–580
Cd	0.1–1.1	4.3×10^{-4}–2.5×10^{-1}	0.0004–0.2
Co	0.03–0.1	2.5×10^{-3}–1.5×10^{0}	0.02–15.0
Cu	0.6–1.6	3.6×10^{-2}–2.1×10^{1}	0.02–13.1
Ni	3–8	7.3×10^{-2}–4.2×10^{1}	0.009–5.2

*: the ratio between estimated concentrations from the waste plume to the highest concentrations in seawater within the CCZ.

SI References

1. Froelich, P. N.; Klinkhammer, G. P.; Bender, M. L.; Luedtke, N. A.; Heath, G. R.; Cullen, D.; Dauphin, P.; Hammond, D.; Hartman, B.; Maynard, V., Early oxidation of organic matter in pelagic sediments of the eastern equatorial Atlantic: suboxic diagenesis. *Geochim. Cosmochim. Acta* **1979,** *43*, (7), 1075-1090.
2. Cutter, G. A.; Moffett, J. W.; Nielsdóttir, M. C.; Sanial, V., Multiple oxidation state trace elements in suboxic waters off Peru: In situ redox processes and advective/diffusive horizontal transport. *Mar. Chem.* **2018,** *201*, 77-89.
3. Rue, E. L.; Smith, G. J.; Cutter, G. A.; Bruland, K. W., The response of trace element redox couples to suboxic conditions in the water column. *Deep Sea Research Part I: Oceanographic Research Papers* **1997,** *44*, (1), 113-134.
4. Moffett, J. W., Iron(II) in the world's oxygen deficient zones. *Chem. Geol.* **2021,** *580*, 120314.
5. Sherrell, R. M.; Twining, B. S.; German, C. R., Trace elements in suspended particles from GO-Flo bottles. In 2016-05-17 ed.; Biological and Chemical Oceanography Data Management Office (BCO-DMO), 2016.
6. Stokes, G. G., On the effect of the internal friction of fluids on the motion of pendulums. *Transactions of the Cambridge Philosophical Society* **1851,** *9*, 8-106.
7. Cowen, J. P.; Massoth, G. J.; Baker, E. T., Bacterial scavenging of Mn and Fe in a mid- to far-field hydrothermal particle plume. *Nature* **1986,** *322*, (6075), 169-171.
8. Buck, K. N.; Sedwick, P. N.; Sohst, B. M.; Carlson, C. A., Organic complexation of iron in the eastern tropical South Pacific: Results from US GEOTRACES Eastern Pacific Zonal Transect (GEOTRACES cruise GP16). *Mar. Chem.* **2018,** *201*, 229-241.
9. Muñoz-Royo, C.; Peacock, T.; Alford, M. H.; Smith, J. A.; Le Boyer, A.; Kulkarni, C. S.; Lermusiaux, P. F. J.; Haley, P. J.; Mirabito, C.; Wang, D.; Adams, E. E.; Ouillon, R.; Breugem, A.; Decrop, B.; Lanckriet, T.; Supekar, R. B.; Rzeznik, A. J.; Gartman, A.; Ju, S. J., Extent of impact of deep-sea nodule mining midwater plumes is influenced by sediment loading, turbulence and thresholds. *Communications Earth & Environment* **2021,** *2*, (1), 148.
10. Stone, A. T. The Reduction and Dissolution of Manganese(III) and (IV) Oxides by Organics. California Institute of Technology, 1983.
11. Stone, A. T.; Morgan, J. J., Reduction and dissolution of manganese(III) and manganese(IV) oxides by organics. 1. Reaction with hydroquinone. *Environ. Sci. Technol.* **1984,** *18*, (6), 450-456.
12. Rubert, K. F.; Pedersen, J. A., Kinetics of oxytetracycline reaction with a hydrous manganese oxide. *Environ. Sci. Technol.* **2006,** *40*, (23), 7216-7221.
13. Herszage, J.; dos Santos Afonso, M.; Luther, G. W., Oxidation of cysteine and glutathione by soluble polymeric MnO2. *Environ. Sci. Technol.* **2003,** *37*, (15), 3332-3338.
14. GEOTRACES Intermediate Data Product Group, The GEOTRACES Intermediate Data Product 2021 (IDP2021). In NERC EDS British Oceanographic Data Centre NOC, 2021.
